# Associations between sex and traditional Chinese medicine body constitutions: the potential role of lifestyle behaviors in a cross-sectional study

**DOI:** 10.3389/fmed.2026.1973268

**Published:** 2026-10-20

**Authors:** Cheng-Hung Lee, Yi-Chang Su, Chia-Ing Li, Chuan-Wei Yang, Tsai-Chung Li

**Affiliations:** 1 Department of Traditional Chinese Medicine, Taichung Veterans General Hospital, Taichung, Taiwan; 2 Department of Post-Baccalaureate Medicine, College of Medicine, National Chung Hsing University, Taichung, Taiwan; 3 National Research Institute of Chinese Medicine, Ministry of Health and Welfare, Taipei, Taiwan; 4 Department of Medical Research, China Medical University Hospital, Taichung, Taiwan; 5 School of Medicine, College of Medicine, China Medical University, Taichung, Taiwan; 6 Department of Public Health, College of Public Health, China Medical University, Taichung, Taiwan; 7 Department of Audiology and Speech-Language Pathology, College of Medical and Health Sciences, Asia University, Taichung, Taiwan

**Keywords:** body constitution, lifestyle, mediation analysis, sex, Traditional Chinese Medicine

## Abstract

**Introduction:**

This study investigated whether smoking, alcohol consumption, and physical inactivity mediate the associations between sex and Traditional Chinese Medicine body constitutions.

**Methods:**

We analyzed data from 35,456 adults aged >18 years enrolled in the Taiwan Biobank. Body constitutions (yang deficiency, yin deficiency, and phlegm-stasis) were assessed using the Body Constitution Questionnaire (BCQ). The BCQ consists of 44 items. Each item is rated on a five-point Likert scale ranging from 1 (“never”) to 5 (“always”), with higher scores indicating a greater tendency toward the corresponding body constitution. Lifestyle behaviors included smoking, alcohol consumption, and leisure-time physical inactivity. Multivariable logistic regression and mediation analyses were performed.

**Results:**

Female sex was independently associated with increased odds of yang deficiency [odds ratio (OR) = 2.52, 95% confidence interval (CI): 2.34–2.71], yin deficiency (OR = 2.09, 95% CI: 1.95–2.25), and phlegm-stasis (OR = 3.03, 95% CI: 2.79–3.30). Smoking showed significant negative indirect association, whereas alcohol consumption exhibited minimal mediation. Physical inactivity statistically accounted for approximately 6% of the observed associations between female sex and all three body constitution patterns.

**Discussion:**

Women were more likely than men to exhibit yang deficiency, yin deficiency, and phlegm-stasis constitutions. Lifestyle behaviors explained only a small proportion of these sex differences. Given the cross-sectional design, these findings should be interpreted as statistical associations rather than evidence of causal mediation.

## Introduction

Traditional Chinese Medicine (TCM) emphasizes individualized treatment based on body constitution, which is determined by the balance between yin and yang (1). Yang represents the energy that supports physiological functions, whereas yin is responsible for nourishing the body through blood and body fluids (1). Disruption of this balance may manifest as symptoms associated with yang or qi deficiency, yin deficiency, or phlegm-stasis stagnation (2–4). Various factors, including aging, chronic diseases, psychological stress, and unhealthy lifestyles, may contribute to constitutional imbalances (2–4). Previous studies have shown that different constitution types are associated with the development and progression of various diseases (4, 5). Accordingly, TCM practitioners tailor treatments to individual constitutional characteristics, even among patients with the same disease diagnosis. This principle, known as “treating the same disease with different therapies,” reflects the personalized approach of TCM and aims to optimize therapeutic outcomes through constitution-based interventions (5, 6).

TCM body constitution is influenced by both congenital and acquired factors. Congenital factors include sex, genetic predisposition, and parental constitution, whereas acquired factors encompass diet, lifestyle, and other environmental influences (7). Sex is considered an important congenital determinant of constitution, as males and females differ in genetic characteristics, body morphology, organ structure, physiological functions, and psychological traits, resulting in constitution-related differences between the sexes. TCM recognizes inherent physiological differences between men and women, which are grounded in the theories of yin–yang balance and the dynamic equilibrium of qi and blood. According to a study in patients with lung adenocarcinoma, men are generally considered to be yang-dominant, whereas women are regarded as yin-dominant (8). Previous empirical findings are consistent with these traditional concepts regarding sex-related physiological differences. A population-based study conducted in Taiwan reported that women were approximately three to four times more likely than men to exhibit yang deficiency, yin deficiency, and phlegm-stasis constitutions, indicating a substantially higher prevalence of unbalanced body constitutions among women (9).

Lifestyle behaviors are increasingly recognized as important determinants of health and may represent a modifiable pathway linking biological sex to TCM body constitutions. Unlike biological sex, which is a non-modifiable characteristic, lifestyle factors such as physical activity, smoking, and alcohol consumption can be modified through behavioral interventions. These behaviors influence metabolic function, immune regulation, inflammatory responses, and psychological wellbeing (10, 11), all of which have been proposed to affect the balance of yin, yang, qi, and blood in TCM theory. Consequently, lifestyle behaviors may partially explain the observed sex differences in body constitutions. A previous study found that smoking was significantly associated with all three biased body constitution patterns, whereas no significant association was observed between alcohol consumption and any of these constitutions (9). Among the lifestyle factors evaluated to date, physical activity has been the most extensively studied in relation to Traditional Chinese Medicine body constitution. Previous studies have consistently reported that regular physical activity is associated with a lower prevalence of biased body constitution patterns than a sedentary lifestyle or the absence of regular exercise (9, 12). Consistent with these findings, research in Taiwan has suggested that physical training may serve as a protective factor against yang deficiency, yin-blood deficiency, and phlegm-stasis constitutions, indicating that maintaining regular exercise habits in patients with breast cancer may help preserve constitutional balance and stability (13). A study's findings indicated that individuals with higher socioeconomic status were more likely to maintain a balanced body constitution, possibly because of more favorable living environments and healthier lifestyles (14). Although these studies have examined the associations between lifestyle factors and Traditional Chinese Medicine body constitution, none has investigated whether lifestyle factors mediate the association between sex and Traditional Chinese Medicine body constitution. Identifying the mediating role of lifestyle behaviors not only enhances understanding of the mechanisms underlying sex-related disparities in TCM body constitutions but also provides potential targets for personalized health promotion and constitution-based preventive strategies.

A mediation model investigates the process through which an independent variable and an outcome variable are interconnected (2). This model allows the identification and illustration of the mechanism or process that elucidates the observed association between an independent variable and an outcome variable by introducing a mediator (2). Unlike assuming a direct causal relationship between the independent variable and the outcome variable, a mediation model suggests that the independent variable influences the mediator, subsequently impacting the outcome variable. This approach aids in comprehending the causal pathway of an association and offers insights into the how and why of the association (4). By elucidating the nature of the relationship between the independent variable and the outcome variable, a mediation model provides a more thorough understanding of the investigated relationship. Incorporating mediators into a study transcends the examination of a straightforward relationship, allowing for a more profound exploration of intricate correlational or causal relationships (4).

Mediation analysis enables the quantification of the extent to which an independent variable conveys the effects of a cause to its corresponding outcome (2, 3). A mediator can elucidate either the entire association or a segment of the observed relationship between two variables. In instances of full mediation, the mediator completely elucidates the relationship between the independent variable and the outcome variable. This means that, when the mediator is incorporated into the model, there is no residual direct relationship between the independent variable and the outcome variable. Conversely, in cases of partial mediation, a statistical relationship persists between the independent variable and outcome variable, even with the inclusion of the mediator in the model. The mediator only partially clarifies the association between the independent variable and the outcome variable. This implies that there is not only a significant relationship between the mediator and the outcome variable but also a direct association between the independent variable and the outcome variable.

In the context of our study, we aim to examine whether lifestyle behaviors act as mediators in the relationship between sex and TCM body constitution. We are interested in determining the extent to which these mediators explain the effects of sex on TCM body constitution. We seek to understand what proportion of the overall effect of sex on TCM body constitution can be attributed to lifestyle behaviors, and what proportion is a direct effect not involving these mediators. This mechanistic perspective allows us to delineate the indirect and direct components of sex's influence on TCM body constitution, taking into account pathways that involve lifestyle behaviors and those that do not.

## Methods

### Participants and study design

This was a cross-sectional study using data from the Taiwan Biobank (TWB). Funded by the Taiwanese government, the TWB is a large, population-based study designed to gather comprehensive environmental, lifestyle, and genomic data from the Taiwanese population. The TWB aims to recruit more than 200,000 community-dwelling healthy individuals aged >18 years with no prior history of cancer. The TWB repository encompasses physical examinations, biochemical profiles (blood and urine assays), multi-omics genetic data (including genotyping and whole-genome sequencing), and structured questionnaires capturing dietary habits and environmental exposures. For the present study, the target population comprised TWB participants aged over 18 years. The inclusion criteria were defined as individuals who (1) were aged over 18 years and (2) self-identified as Taiwanese Han Chinese. Figure 1 illustrates the participant selection process. A total of 200,661 participants were initially enrolled in the Taiwan Biobank. Of these, 164,512 were excluded because of missing data from the Body Constitutions Questionnaire, and an additional 693 participants were excluded due to incomplete information on sociodemographic characteristics, lifestyle behaviors, disease history, or biochemical indices. Consequently, 36,149 participants met the eligibility criteria, and 35,456 participants were ultimately included in the final analysis. Key demographic characteristics and relevant study variables were compared using standardized mean differences (SMDs), which allow assessment of the magnitude of differences between groups independent of sample size. The results of this comparison are now presented in Supplementary Table S1. The SMDs were small for most characteristics, with the exception of marital status (SMD = 0.13), which indicated only a small difference between the two groups. Overall, these findings suggest that the included and excluded participants were generally similar in their observed characteristics and provide no indication of substantial selection bias based on the variables examined. The TWB protocol was conducted in strict accordance with relevant ethical and legal standards, and all participants provided written informed consent prior to enrollment. All research procedures adhered to applicable regulatory guidelines to ensure participant privacy and data security. The IRB of this study was approved by the Institutional Review Board of Taichung Veterans General Hospital, Taiwan (IRB TCVGH No: CE25620B).

**Figure 1:**
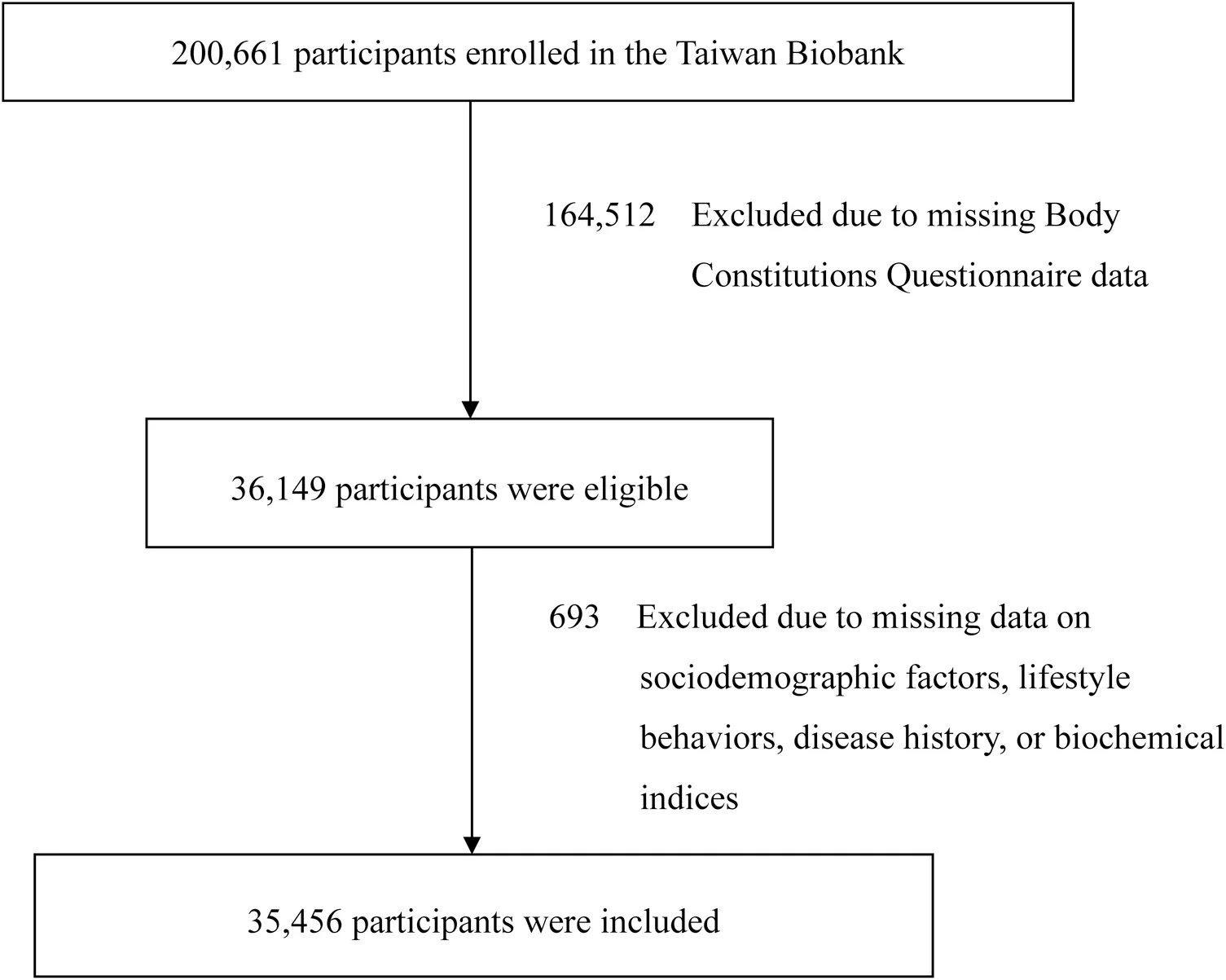
Flowchart of procedures for defining eligible study subjects.

### Measurements

Sociodemographic characteristics included age, educational level, and marital status. Lifestyle behaviors comprised cigarette smoking, alcohol consumption, and leisure-time physical activity. Smoking status was categorized as never, former, or current smoker according to participants’ smoking history. Never smokers were those who had never smoked. Former smokers were those who had smoked regularly for at least 6 months but had subsequently quit, whereas current smokers were those who had smoked regularly for at least 6 months and continued to smoke at the time of data collection. For the primary analyses, former and current smokers were combined as ever smokers, with never smokers serving as the reference group. Alcohol consumption was categorized as never/seldom, former, or current drinking based on self-reported drinking status. Among current and former drinkers, alcohol intake was recorded as the volume of alcoholic beverages consumed per week. Regular alcohol consumption was defined according to the Taiwan Biobank questionnaire as consumption of more than 150 mL/week for at least 6 months. Because the available measure represented beverage volume rather than ethanol content, alcohol exposure was expressed as mL/week and was not converted to grams of ethanol or standard drinks. The duration since cessation was not available for former drinkers. Leisure-time physical activity was assessed based on the frequency and duration of exercise. Participants who reported exercising at least three times per week for more than 30 min per session were classified as physically active; all other participants were classified as physically inactive. Thus, the physically inactive category included participants who did not meet this predefined frequency and duration criterion and did not necessarily indicate complete absence of physical activity. Anthropometric measurements included waist circumference, hip circumference, body mass index (BMI), and waist-to-hip ratio (WHR). Laboratory measurements included low-density lipoprotein cholesterol (LDL-C), high-density lipoprotein cholesterol (HDL-C), total cholesterol, triglycerides, fasting plasma glucose (FPG), glycated hemoglobin (HbA1c), creatinine, estimated glomerular filtration rate (eGFR), blood urea nitrogen (BUN), and uric acid. All laboratory analyses were performed at the Department of Clinical Laboratory, Linkou Chang Gung Memorial Hospital. FPG, lipid profiles, creatinine, albumin, BUN, and microalbumin were analyzed using the Hitachi LST 008 analyzer (Hitachi High-Tech Corporation, Tokyo, Japan), whereas uric acid was measured using the Abbott Architect i2000 analyzer (Abbott Laboratories, Abbott Park, IL, USA). Comorbidities included hypertension, diabetes mellitus, hyperlipidemia, stroke, valvular heart disease, coronary artery disease, arrhythmia, osteoporosis, arthritis, gout, asthma, gastroesophageal reflux disease, and irritable bowel syndrome.

### Body constitutions

Body constitution was assessed using the Body Constitution Questionnaire (BCQ), a validated self-administered instrument developed by Su et al. based on the theoretical framework of TCM (15). The BCQ evaluates an individual's constitutional characteristics by measuring three constitution types: yang deficiency, yin deficiency, and phlegm-stasis. Yang deficiency reflects insufficient functional energy and is characterized by reduced vitality and intolerance to cold. Yin deficiency represents inadequate body fluids and nourishment, typically presenting with symptoms of dryness and heat intolerance. Phlegm-stasis reflects impaired circulation and the accumulation of pathological substances, which may manifest as fatigue, heaviness, or discomfort. BCQ is well developed and validated (6, 15, 16). Through a two-stage Delphi process, 44 items of the BCQ were generated, with initial items being translated into colloquial questions. The BCQ included 19 items for yang deficiency, 19 items for yin deficiency, and 16 items for phlegm-stasis, with some items shared across constitution domains. Each item in the BCQ was tested for wording, sequencing, grammar correctness, and easy comprehension. The number of BCQ items was reduced through the reliability evaluation of intraclass consistency (6, 15, 16). Prior studies demonstrated that the BCQ had favorable factorial validity (6), and the Cronbach's *α* of each constitution subscale ranged from 0.88 to 0.90 (5, 6, 16). The BCQ consists of 44 items, including 19 items for yang deficiency, 19 items for yin deficiency, and 16 items for phlegm-stasis, with some items shared across constitution domains. Each item is rated on a five-point Likert scale ranging from 1 (“never”) to 5 (“always”), with higher scores indicating a greater tendency toward the corresponding body constitution. According to the recommended cutoff values, yang deficiency was defined as a total score ≥31, yin deficiency as a total score ≥30, and phlegm-stasis as a total score ≥27 (5, 16). Participants may simultaneously meet the criteria for more than one body constitution. The BCQ has demonstrated good reliability and validity in previous studies and has been widely used to assess body constitution in Taiwanese populations (5). The three constitution outcomes were not mutually exclusive. Participants could simultaneously meet the diagnostic criteria for more than one constitution. Accordingly, yang deficiency, yin deficiency, and phlegm-stasis were treated as three separate, potentially overlapping binary outcomes, and each outcome was analyzed independently in the logistic regression and mediation analyses.

### Statistical analysis

Descriptive statistics were presented as means and standard deviations (SDs) for continuous variables, and two-sample *t*-tests were used to compare continuous variables between groups. Categorical variables were presented as frequencies and percentages, and chi-square tests were used for comparisons between groups. Multivariable logistic regression analyses were performed to examine the associations between individual covariates and the outcome variables. Models were adjusted for prespecified covariates encompassing sociodemographic characteristics (age, education, and marital status), lifestyle behaviors (smoking, alcohol consumption, and physical activity), clinical conditions (hypertension, diabetes, hyperlipidemia, stroke, valvular heart disease, coronary artery disease, osteoporosis, arthritis, gout, and irritable bowel syndrome), anthropometric measures (BMI and waist-to-hip ratio), and metabolic and laboratory measures [systolic blood pressure, diastolic blood pressure, fasting plasma glucose, HbA1c, triglycerides, alanine aminotransferase (ALT), and eGFR]. These variables were treated as adjustment covariates rather than being assumed to represent a uniform set of causal confounders. Given that the analyses addressed several prespecified research questions involving different outcomes and lifestyle behaviors, we did not apply a single global multiple-comparison adjustment across all statistical tests. Statistical inference was therefore based primarily on the magnitude and precision of the estimated associations, as reflected by effect estimates and 95% confidence intervals (CIs), rather than statistical significance alone. All statistical analyses were performed using SAS version 9.4 (SAS Institute Inc., Cary, NC, USA). All *p*-values were two-sided, and *p* < 0.05 was considered statistically significant.

Effect modification was evaluated separately from the mediation analyses. Whereas mediation analysis examined whether lifestyle behaviors statistically accounted for part of the association between sex and TCM body constitutions, effect modification analysis assessed whether the magnitude of the association between sex and each body constitution differed according to lifestyle behavior status. Thus, mediation and interaction represent distinct analytical concepts and were interpreted separately. For effect modification analyses, associations between sex and each of the three body constitution outcomes were estimated within strata of smoking status, alcohol consumption, and physical activity. Multiplicative interaction terms between sex and each lifestyle behavior were included in the corresponding multivariable logistic regression models, and the statistical significance of the interaction term was evaluated using the *p*-value for interaction. Stratified odds ratios (ORs) and their 95% confidence intervals were reported to facilitate interpretation of significant interactions. Because the three body constitution outcomes were potentially overlapping, interaction analyses were conducted separately for yang deficiency, yin deficiency, and phlegm-stasis.

A directed acyclic graph (DAG) depicting a model of the mediating effect of lifestyle behaviors on the relationship between sex and TCM body constitutions is shown in Supplementary Figure S1. Mediation analyses were conducted to evaluate whether smoking, alcohol consumption, and leisure-time physical inactivity statistically accounted for part of the associations between sex and TCM body constitutions. The mediation analysis was conceptually guided by the framework proposed by Baron and Kenny (17). Because the TCM body constitution outcomes were dichotomized, logistic regression models with a logit link were used throughout the analyses. First, the total association between sex and each TCM body constitution was estimated using logistic regression. Second, the associations between sex and each potential mediator (smoking, alcohol consumption, and leisure-time physical inactivity) were examined. Third, each TCM body constitution was regressed simultaneously on sex and the corresponding lifestyle behavior to assess the association between the potential mediator and the body constitution after adjustment for sex, and to determine whether the estimated association between sex and the body constitution was attenuated after inclusion of the potential mediator. All models were adjusted for the prespecified covariates described earlier. The total, direct, and indirect effects were estimated within the logistic regression-based mediation analysis with a counterfactual-based causal mediation framework implemented in SAS PROC CAUSALMED. The outcome models were specified using logistic regression with a binomial distribution and logit link. Indirect effects were assessed using non-parametric bootstrapping (18), and 95% bootstrap CIs were calculated. Statistical significance of an indirect effect was determined when its 95% CI did not include zero. Effect estimates were expressed as ORs with corresponding 95% CIs, where appropriate. Because this study used cross-sectional data, the mediation analyses were interpreted as statistical mediation or indirect associations rather than as evidence of causal mediation or a temporal causal pathway. We conducted a sensitivity analysis using multiple imputation to replace missing values among participants who would otherwise have been excluded because of missing BCQ information and/or covariates. The mediation analyses were then repeated using the imputed datasets. The other sensitivity analysis was performed using modified Poisson regression with robust variance to estimate prevalence ratios (PRs) and their corresponding 95% CI.

## Results

Table 1 summarizes the baseline characteristics of the study population by sex. Compared with women, men were older, had higher educational attainment, and were more likely to smoke, consume alcohol, engage in regular physical activity, and have cardiometabolic comorbidities, including hypertension, diabetes, hyperlipidemia, coronary artery disease, and gout (all *p* < 0.001). Men also exhibited higher BMI, waist circumference, blood pressure, FPG, HbA1c, triglycerides, liver enzyme levels, serum creatinine, and LDL-C, whereas women had higher total cholesterol, HDL-C, and eGFR (all *p* < 0.001). No significant sex differences were found for arrhythmia, asthma, or gastroesophageal reflux disease.

**Table 1:** Baseline characteristics of the study population grouped by sex.

Variables	Total (*n* = 35,456)	Sex *N* (%)		*p*-value
		Male (*n* = 13,961)	Female (*n* = 21,495)	
Sociodemographic factors				
Age (year)^a^	49.47 ± 11.86	49.91 ± 12.31	49.18 ± 11.55	<0.001
Education level				<0.001
≤6 years	914 (2.58)	221 (1.58)	693 (3.22)	
7–12 years	10,834 (30.56)	3,432 (24.58)	7,402 (34.44)	
>12 years	23,708 (66.87)	10,308 (73.83)	13,400 (62.34)	
Marriage				<0.001
Unmarried	6,120 (17.26)	2,168 (15.53)	3,952 (18.39)	
Married	25,338 (71.46)	10,892 (78.02)	14,446 (67.21)	
Separated	2,715 (7.66)	738 (5.29)	1,977 (9.2)	
Widowed	1,283 (3.62)	163 (1.17)	1,120 (5.21)	
Lifestyle behaviors				
Smoking				<0.001
Never	29,256 (82.51)	8,701 (62.32)	20,555 (95.63)	
Former	3,562 (10.05)	3,082 (22.08)	480 (2.23)	
Current	2,638 (7.44)	2,178 (15.60)	460 (2.14)	
Drinking				<0.001
Never	32,104 (90.55)	11,307 (80.99)	20,797 (96.75)	
Former	961 (2.71)	786 (5.63)	175 (0.81)	
Current	2,391 (6.74)	1,868 (13.38)	523 (2.43)	
Physical activity				<0.001
Inactive	21,286 (60.03)	7,893 (56.54)	13,393 (62.31)	
Active	14,170 (39.97)	6,068 (43.46)	8,102 (37.69)	
Disease history				
Hypertension	4,186 (11.81)	2,329 (16.68)	1,857 (8.64)	<0.001
Diabetes	1,855 (5.23)	999 (7.16)	856 (3.98)	<0.001
Hyperlipidemia	2,959 (8.35)	1,442 (10.33)	1,517 (7.06)	<0.001
Stroke	238 (0.67)	160 (1.15)	78 (0.36)	<0.001
Valvular heart disease	1,577 (4.45)	403 (2.89)	1,174 (5.46)	<0.001
Coronary artery disease	490 (1.38)	359 (2.57)	131 (0.61)	<0.001
Arrhythmia	260 (0.73)	655 (4.69)	983 (4.57)	0.62
Osteoporosis	1,482 (4.18)	343 (2.46)	1,139 (5.3)	<0.001
Arthritis	1,789 (5.05)	539 (3.86)	1,250 (5.82)	<0.001
Gout	1,428 (4.03)	1,304 (9.34)	124 (0.58)	<0.001
Asthma	1,397 (3.94)	577 (4.13)	820 (3.81)	0.13
GERD	5,379 (15.17)	2,068 (14.81)	3,311 (15.40)	0.13
IBS	1,115 (3.14)	479 (3.43)	636 (2.96)	0.01
Biomarker^a^				
BMI (kg/m^2^)	24.24 ± 3.84	25.34 ± 3.60	23.52 ± 3.83	<0.001
Waist (cm)	83.46 ± 10.42	88.02 ± 9.60	80.50 ± 9.86	<0.001
Hip (cm)	96.35 ± 7.24	97.78 ± 6.83	95.42 ± 7.35	<0.001
WHR	0.86 ± 0.07	0.90 ± 0.06	0.84 ± 0.07	<0.001
SBP (mmHg)	118.71 ± 17.92	124.57 ± 16.48	114.91 ± 17.79	<0.001
DBP (mmHg)	73.83 ± 11.27	78.15 ± 10.90	71.02 ± 10.60	<0.001
FPG (mg/dL)	95.99 ± 20.07	99.13 ± 21.88	93.94 ± 18.51	<0.001
HbA1c (%)	5.76 ± 0.78	5.83 ± 0.84	5.71 ± 0.73	<0.001
TG (mg/dL)	114.88 ± 92.79	134.80 ± 113.97	101.93 ± 73.08	<0.001
TC (mg/dL)	196.34 ± 36.30	192.69 ± 35.94	198.71 ± 36.34	<0.001
HDL-C (mg/dL)	55.61 ± 13.99	49.22 ± 11.50	59.77 ± 13.90	<0.001
LDL-C (mg/dL)	120.24 ± 32.04	121.14 ± 32.18	119.66 ± 31.93	<0.001
AST (U/L)	22.86 ± 11.89	24.68 ± 13.15	21.68 ± 10.84	<0.001
ALT(U/L)	24.60 ± 22.01	30.32 ± 26.15	20.89 ± 17.88	<0.001
SCr (mg/dL)	0.74 ± 0.67	0.92 ± 1.00	0.63 ± 0.22	<0.001
eGFR (mL/min/1.73 m^2^)	101.56 ± 15.26	96.35 ± 15.77	104.94 ± 13.91	<0.001

GERD, gastroesophageal reflux disease; IBS, irritable bowel syndrome; BMI, body mass index; WHR, waist-to-hip ratio; SBP, systolic blood pressure; DBP, diastolic blood pressure; FPG, fasting plasma glucose; HbA1c, hemoglobin A1c; TG, triglyceride; TC, total cholesterol; HDL-C, high-density lipoprotein cholesterol; LDL, low-density lipoprotein; AST, aspartate aminotransferase; ALT, alanine aminotransferase; SCr, serum creatinine; eGFR, estimated glomerular filtration rate.

Differences in continuous variables were tested using Student's *t* test. Differences in categorical variables were tested using the chi-square test.

a Mean ± SD.

Table 2 compares body constitution scores and the prevalence of body constitution patterns between men and women. The prevalence of all three body constitution patterns was also higher among women than men: Yang deficiency was present in 29.1% of women versus 15.1% of men, corresponding to an absolute difference of 14.0 percentage points; yin deficiency was present in 28.5% versus 18.4%, corresponding to a difference of 10.2 percentage points; and phlegm-stasis was present in 22.6% versus 11.3%, corresponding to a difference of 11.4 percentage points (all *p* < 0.001).

**Table 2:** Comparison of body constitutions by sex.

Body constitution	Total	Sex *N* (%)		*p*-value
		Male (*n* = 13,961)	Female (*n* = 21,495)	
Yang deficiency	27.23 ± 6.72	25.62 ± 5.66	28.28 ± 7.14	<0.001
No	27,085 (76.39)	11,852 (84.89)	15,233 (70.87)	
Yes	8,371 (23.61)	2,109 (15.11)	6,262 (29.13)	
Yin deficiency	26.53 ± 6.26	25.41 ± 5.51	27.26 ± 6.61	<0.001
No	26,764 (75.49)	11,399 (81.65)	15,365 (71.48)	
Yes	8,692 (24.51)	2,562 (18.35)	6,130 (28.52)	
Phlegm-stasis	22.20 ± 5.91	20.79 ± 4.87	23.11 ± 6.34	<0.001
No	29,019 (81.85)	12,391 (88.75)	16,628 (77.36)	
Yes	6,437 (18.15)	1,570 (11.25)	4,867 (22.64)	

Table 3 shows that female sex was independently associated with increased odds of yang deficiency (OR = 2.52, 95% CI: 2.34–2.71), yin deficiency (OR = 2.09, 95% CI: 1.95–2.25), and phlegm-stasis (OR = 3.03, 95% CI: 2.79–3.30). Smoking and physical inactivity were also significantly associated with all three body constitution patterns, whereas alcohol consumption showed weaker associations and was not significantly associated with yin deficiency. These findings remained consistent after adjustment for sociodemographic characteristics, comorbidities, anthropometric measures, and metabolic biomarkers. Compared with the age-adjusted models, the magnitude of the associations between female sex and the three body constitution patterns was modestly attenuated after adjustment for smoking, alcohol consumption, and physical activity, although the associations remained highly significant.

**Table 3:** Associations of sex and lifestyle behaviors with body constitutions.

Variables	OR (95% CI)			
	Female (male as reference)	Mediator: smoking (never as reference)	Mediator: drinking (never as reference)	Mediator: physical activity (active as reference)
Yang deficiency				
Age-adjusted	2.49 (2.34, 2.65)***	1.22 (1.13, 1.33)***	1.16 (1.05, 1.28)**	1.47 (1.39, 1.56)***
Multivariate-adjusted^a^	2.59 (2.43, 2.77)***	1.25 (1.15, 1.36)***	1.17 (1.06, 1.29)**	1.49 (1.41, 1.58)***
Multivariate-adjusted^b^	2.52 (2.34, 2.71)***	1.24 (1.14, 1.34)***	1.16 (1.05, 1.28)**	1.49 (1.40, 1.58)***
Yin deficiency				
Sex, age-adjusted	1.93 (1.82, 2.05)***	1.30 (1.21, 1.41)***	1.10 (1.00, 1.20)	1.44 (1.37, 1.52)***
Multivariate-adjusted^a^	2.01 (1.89, 2.14)***	1.32 (1.22, 1.43)***	1.09 (1.00, 1.20)	1.46 (1.38, 1.54)***
Multivariate-adjusted^b^	2.09 (1.95, 2.25)***	1.30 (1.20, 1.41)***	1.09 (0.99, 1.20)	1.44 (1.37, 1.53)***
Phlegm-stasis				
Sex, age-adjusted	2.60 (2.42, 2.79)***	1.39 (1.27, 1.52)***	1.18 (1.06, 1.31)**	1.63 (1.53, 1.74)***
Multivariate-adjusted^a^	2.75 (2.56, 2.96)***	1.42 (1.30, 1.56)***	1.17 (1.05, 1.31)**	1.65 (1.55, 1.76)***
Multivariate-adjusted^b^	3.03 (2.79, 3.30)***	1.38 (1.26, 1.52)***	1.16 (1.04, 1.30)**	1.60 (1.50, 1.70)***

Data are presented as odds ratio (95% confidence interval).

a Multivariate-adjusted for age, education, marriage, smoking, alcohol consumption, leisure-time physical activity, hypertension, diabetes, hyperlipidemia, stroke, valvular heart disease, coronary artery disease, osteoporosis, arthritis, gout, and IBS.

b Multivariate-adjusted for age, education, marriage, smoking, alcohol consumption, leisure-time physical activity, hypertension, diabetes, hyperlipidemia, stroke, valvular heart disease, coronary artery disease, osteoporosis, arthritis, gout, IBS, BMI, WHR, SBP, DBP, FPG, HbA1c, TC, TG, ALT, and eGFR.

** *p* < 0.01; ***p < 0.001.

Figure 2 presents the associations between sex and the three body constitution types stratified by lifestyle behaviors. Female sex was consistently associated with increased odds of yang deficiency, yin deficiency, and phlegm-stasis regardless of smoking, alcohol consumption, or physical activity status, with all associations remaining statistically significant (all *p* < 0.001). Significant interactions between sex and smoking were observed for yin deficiency (*p* for interaction = 0.002) and phlegm-stasis (*p* for interaction = 0.003), indicating that the associations between sex and these body constitution types differed according to smoking status. In contrast, no significant interactions were found for yang deficiency (*p* = 0.07) or for alcohol consumption and physical activity across any of the three body constitution types (all *p* for interaction > 0.05).

**Figure 2:**
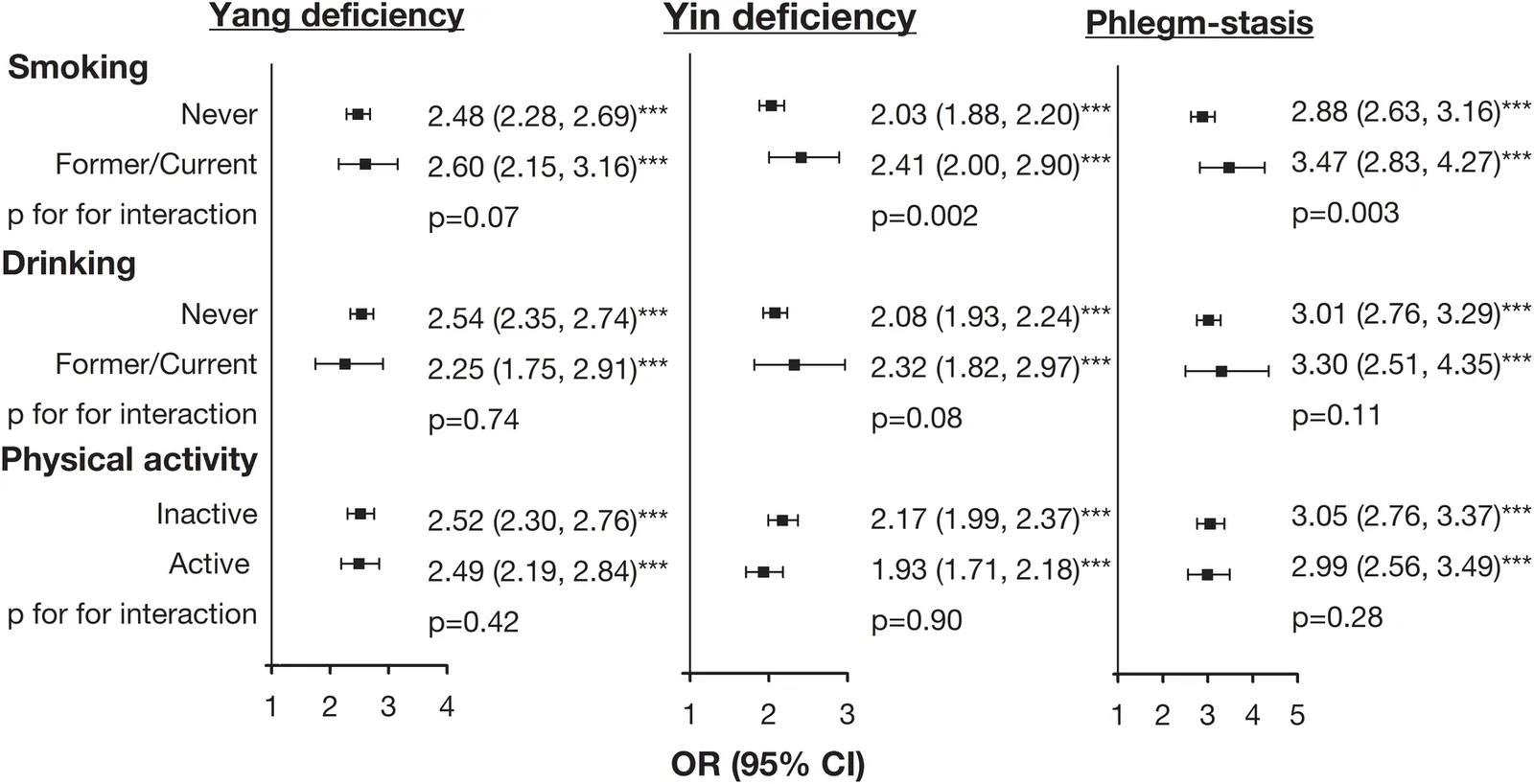
Associations between sex and TCM body constitutions stratified by lifestyle behaviors and tests for interaction. ***p < 0.001.

Table 4 presents the mediation analysis examining the associations between sex and the three body constitution types through smoking, alcohol consumption, and physical inactivity. Female sex remained significantly associated with higher odds of yang deficiency, yin deficiency, and phlegm-stasis across all models, with direct associations consistently exceeding the total effects when smoking or alcohol consumption was considered as the mediator. Smoking exhibited a significant negative indirect association on all three body constitutions (indirect effect ORs ranging from 0.90 to 0.94), corresponding to negative proportions mediated of 12.00% for Yang deficiency, 18.26% for Yin deficiency, and 17.59% for phlegm-stasis, indicating an indirect association in the opposite direction to the total association. Similarly, alcohol consumption demonstrated only minimal negative mediation, with proportions mediated ranging from 1.25% to 1.93%, although the indirect association was not statistically significant for yin deficiency. In contrast, physical inactivity significantly and positively mediated the associations between female sex and all three body constitution types (indirect effect ORs = 1.04–1.05), accounting for 6.23%, 6.64%, and 6.53% of the total effects for yang deficiency, yin deficiency, and phlegm-stasis, respectively. The results for the sensitivity analysis with multiple imputation are presented in Supplementary Table S2. The results obtained from the multiple-imputation analyses were generally consistent with those of the complete-case analyses, supporting the robustness of our findings to potential bias related to missing data. The results for the sensitivity analysis with modified Poisson regression with robust variance were generally consistent with those of the primary logistic regression analyses, with the direction and statistical significance of the associations remaining similar (Supplementary Table S3).

**Table 4:** Associations of sex and body constitutions with lifestyle behaviors as mediators.

Outcome	OR (95% CI)^d^			Proportion mediated (%)^c^
	Total effect^a^	Direct effect^b^	Indirect effect	
Yang deficiency				
Mediator: smoking	2.35 (2.19, 2.52)***	2.52 (2.33, 2.70)***	0.94 (0.91, 0.96)***	−12.00 (−17.29, −6.72)***
Mediator: drinking	2.49 (2.30, 2.67)***	2.52 (2.33, 2.70)***	0.99 (0.98, 1.00)**	−1.93 (−3.32, −0.54)***
Mediator: physical inactivity	2.62 (2.42, 2.81)***	2.52 (2.33, 2.70)***	1.04 (1.03, 1.05)***	6.23 (5.01, 7.45)***
Yin deficiency				
Mediator: smoking	1.92 (1.80, 2.05)***	2.09 (1.94, 2.24)***	0.92 (0.89, 0.94)***	−18.26 (−24.63, −11.89)***
Mediator: drinking	2.08 (1.93, 2.22)***	2.09 (1.94, 2.24)***	0.99 (0.99, 1.00)	−1.25 (−2.67, 0.18)
Mediator: physical inactivity	2.17 (2.02, 2.32)***	2.09 (1.94, 2.24)***	1.04 (1.03, 1.04)***	6.64 (5.29, 8.00)***
Phlegm-stasis				
Mediator: smoking	2.73 (2.51, 2.94)***	3.03 (2.78, 3.29)***	0.90 (0.87, 0.93)***	−17.59 (−23.45, −11.73)***
Mediator: drinking	3.00 (2.75, 3.25)***	3.03 (2.78, 3.29)***	0.99 (0.98, 1.00)***	−1.72 (−3.09, −0.35)***
Mediator: physical inactivity	3.17 (2.91, 3.44)***	3.03 (2.78, 3.29)***	1.05 (1.04, 1.06)***	6.53 (5.29, 7.76)***

a Adjusted for age, education, marriage, hypertension, diabetes, hyperlipidemia, stroke, valvular heart disease, coronary artery disease, osteoporosis, arthritis, gout, IBS, BMI, WHR, SBP, DBP, FPG, HbA1c, TC, TG, ALT, eGFR, and sex.

b Adjusted for age, education, marriage, hypertension, diabetes, hyperlipidemia, stroke, valvular heart disease, coronary artery disease, osteoporosis, arthritis, gout, IBS, BMI, WHR, SBP, DBP, FPG, HbA1c, TC, TG, ALT, eGFR, sex, and the mediators of interest (smoking, alcohol consumption or physical activity).

c The proportion mediated was calculated using the formula: (Natural indirect effect/Total effect) × 100.

d Percentile-based bootstrap CIs are reported.

** *p* < 0.01; ***p < 0.001.

## Discussion

The present study demonstrated that women were more likely than men to exhibit yang deficiency, yin deficiency, and phlegm-stasis constitutions, and these associations remained robust after adjustment for a wide range of potential confounders. Although smoking, alcohol consumption, and physical inactivity were all associated with body constitution patterns, adjustment for these lifestyle behaviors only modestly attenuated the associations between sex and body constitutions. To our knowledge, this is the first study to investigate whether lifestyle behaviors mediate the associations between sex and TCM body constitution patterns. Our mediation analyses demonstrated that physical inactivity partially mediated the associations between female sex and all three body constitution patterns. In contrast, smoking and alcohol consumption exhibited negative indirect association. Overall, these findings suggest that lifestyle behaviors account for only a modest proportion of the observed sex differences in TCM body constitutions.

The indirect associations through lifestyle behaviors differed in direction and magnitude. Physical inactivity showed a positive indirect association and statistically accounted for approximately 6%–7% of the observed associations between female sex and the three TCM body constitutions. In contrast, smoking showed negative indirect associations, resulting in negative proportions mediated. This pattern indicates that the indirect association through smoking operated in the opposite direction to the overall association. One possible explanation is that smoking was less prevalent among women than men in this population; however, given the cross-sectional design, this finding should be interpreted as a statistical indirect association rather than as evidence that smoking causally mediates the association between sex and TCM body constitution. The underlying mechanisms remain uncertain and warrant investigation in longitudinal studies.

The effect estimates changed modestly after adjustment for demographic and lifestyle-related covariates. For example, the OR for yang deficiency increased from 2.49 in the age-adjusted model to 2.59 after additional adjustment and subsequently decreased to 2.52 in the fully adjusted model. These changes indicate that the covariates influenced the estimated association between sex and TCM body constitutions. Although the initial increase may be compatible with negative confounding or suppression, changes in logistic regression odds ratios after adjustment can also reflect the non-collapsibility of the odds ratio. Therefore, these changes should not be interpreted as definitive evidence of a specific confounding mechanism. Importantly, the overall magnitude and direction of the associations remained broadly consistent across the models.

Direct comparison of the magnitude of the observed associations with previous studies is challenging because most prior studies of sex differences in TCM body constitutions have primarily reported prevalence or constitution scores rather than comparable effect estimates. Nevertheless, variation in the magnitude of sex differences across studies may reflect differences in participant characteristics, including age and demographic composition, as well as differences in BCQ administration, cutoff criteria, and statistical methods. The present study used prespecified BCQ thresholds and multivariable logistic regression in a large sample, providing adjusted estimates of the association between sex and each constitution pattern. However, the absence of standardized effect estimates in the existing literature limits quantitative comparisons and highlights the need for future studies to report both absolute prevalence differences and effect estimates with confidence intervals.

TCM recognizes inherent physiological differences between men and women, which are rooted in the principles of Yin–Yang theory and the dynamic balance of qi and blood. According to TCM theory, men are generally characterized by a predominance of yang, whereas women tend to exhibit a predominance of yin, resulting in distinct body constitution profiles. Consistent with this theoretical framework, a previous Taiwan Biobank study involving 2,760 adults reported that women were approximately three to four times more likely than men to present with yang deficiency, yin deficiency, and phlegm-stasis constitutions (9). Our findings are in agreement with this sex-specific pattern; however, the estimated associations were somewhat attenuated, with women demonstrating approximately two- to three-fold higher odds of these constitution types. The discrepancy in effect size may be attributable to differences in study populations, sample size, participant characteristics, or statistical adjustment for potential confounding factors.

Although TCM clinical practice advocates individualized treatment according to body constitution, epidemiological evidence on constitution-related determinants remains scarce. In the present study, physical inactivity was independently associated with yang deficiency, yin deficiency, and phlegm-stasis constitutions, with the strongest association observed for the phlegm-stasis constitution. Our findings are consistent with those of a nationwide study of breast cancer survivors, which demonstrated that regular physical activity was associated with a lower prevalence of combined yang deficiency, yin deficiency, and phlegm-stasis constitutions and may help maintain constitutional balance (13). However, another study reported that physical activity was significantly associated only with the phlegm-stasis constitution. This discrepancy may be partly explained by the relatively small sample size of that study (*n* = 2,760), which may have limited its statistical power to detect associations with other constitution types (9). From a TCM perspective, regular exercise is believed to promote the circulation of qi and blood, strengthen yang qi, and prevent the accumulation of phlegm and stasis. These mechanisms may partly explain the observed protective association between physical activity and constitution balance in the present study.

The present findings have several clinical implications. Women had a higher prevalence of yang deficiency, yin deficiency, and phlegm-stasis constitutions and may therefore benefit from earlier assessment and closer monitoring of body constitution in both clinical practice and health promotion programs. Although physical inactivity explained only a modest proportion of the associations between sex and body constitution patterns, it represents a modifiable factor that could be targeted through lifestyle interventions, particularly among women. The limited mediating effects observed in this study further suggest that lifestyle modification alone is unlikely to fully account for sex-related differences in body constitutions. Accordingly, individualized and sex-specific approaches that integrate lifestyle counseling with consideration of biological and psychosocial factors may be more effective in promoting constitutional balance. Given the reported associations between unbalanced body constitutions and subsequent chronic diseases, early identification of susceptible individuals and targeted preventive interventions may also contribute to reducing future disease risk.

Clinicians should not assume that the higher prevalence of TCM body constitution patterns among women can be explained solely by the lifestyle behaviors examined in this study. The remaining association may reflect other unmeasured biological, behavioral, social, or psychosocial factors, including dietary factors, socioeconomic circumstances, sleep, occupation, reproductive or menopausal factors, medication use, healthcare utilization, or other sources of residual confounding. Further studies incorporating a broader range of relevant factors are warranted to better understand the observed sex differences in TCM body constitutions.

A major strength of this study is the large sample size derived from the Taiwan Biobank, which provided sufficient statistical power to detect relatively modest associations and yielded precise effect estimates. The availability of detailed information on sociodemographic characteristics, lifestyle behaviors, anthropometric measurements, comorbidities, and metabolic biomarkers allowed comprehensive adjustment for potential confounding factors. Another strength of this study is the standardized data collection implemented by the Taiwan Biobank, which enhanced the reliability and consistency of the measurements.

Several limitations should be noted. First, the cross-sectional design precludes causal inference and limits the interpretation of the observed mediation effects as causal mechanisms. Although mediation analysis was used to examine whether lifestyle behaviors statistically accounted for part of the association between sex and TCM body constitutions, the cross-sectional data do not establish the temporal ordering among sex, lifestyle behaviors, and body constitutions. Therefore, the observed indirect effects should not be interpreted as evidence of causal mediation or as confirmation of a causal pathway. Reverse or bidirectional associations between lifestyle behaviors and TCM body constitutions cannot be excluded. Future longitudinal studies incorporating repeated and, where feasible, objective assessments of lifestyle behaviors and TCM body constitutions are warranted to clarify temporal and potentially causal relationships. Second, lifestyle behaviors and TCM body constitutions were assessed using self-reported questionnaires, which may have introduced recall and reporting bias and potentially resulted in exposure or outcome misclassification. Third, because Taiwan Biobank participants were volunteers, the findings may not be fully generalizable to the general population. Fourth, the representativeness and external validity of the study population are limited. Although the Taiwan Biobank provides a large-scale resource for population health research, its participants are volunteers and therefore do not constitute a fully representative probability sample of the Taiwanese population. In addition, the present analysis was restricted to Taiwanese Han Chinese adults aged >18 years. Therefore, our findings should primarily be interpreted as applicable to Taiwanese Han Chinese adults aged >18 years included in the analyzed Taiwan Biobank sample and should not be directly generalized to individuals younger than 18 years, other ethnic groups, Asian populations in general, or populations outside Taiwan. Further studies in more diverse and representative populations are warranted to determine whether these observed sex differences and the potential role of lifestyle behaviors are consistent across different demographic and ethnic groups. Fifth, given the number of statistical comparisons performed, the possibility of chance findings due to multiple testing cannot be completely excluded. In addition, the large sample size may result in statistically significant *p*-values even for relatively small effect sizes. Therefore, the findings should be interpreted primarily according to the magnitude and precision of the associations and their potential clinical or public health relevance rather than statistical significance alone. Finally, the covariate adjustment strategy was limited. Although we adjusted for a broad range of sociodemographic, lifestyle, clinical, anthropometric, and metabolic characteristics, the cross-sectional design does not allow the temporal or causal ordering of these variables to be established. Some adjusted variables, such as BMI, metabolic biomarkers, and clinical conditions, may potentially lie downstream of lifestyle behaviors, and their inclusion could result in overadjustment. Conversely, conditioning on variables that may act as colliders under certain causal structures could introduce collider bias. Therefore, the fully adjusted estimates should be interpreted as adjusted statistical associations rather than causal effects, and residual confounding or bias due to the covariate adjustment strategy cannot be completely excluded.

## Conclusion

Women were more likely than men to exhibit yang deficiency, yin deficiency, and phlegm-stasis constitutions. Lifestyle behaviors explained only a small proportion of these sex differences. Physical inactivity statistically accounted for the observed associations between female sex and all three body constitution patterns. Given the cross-sectional design, these findings should be interpreted as statistical associations rather than evidence of causal mediation. Further studies are warranted to clarify these mechanisms and support the development of sex-specific prevention and management strategies.

## Data Availability

The datasets presented in this article are not readily available because access to the Taiwan Biobank dataset is restricted and requires approval from the Taiwan Biobank. The data may only be used for approved research purposes and must not be used to identify or re-identify individual participants or shared with unauthorized third parties. Requests to access the datasets should be directed to the Taiwan Biobank website: https://www.twbiobank.org.tw/.
