# Supplementary material for: Associations between sex and traditional Chinese medicine body constitutions: the potential role of lifestyle behaviors in a cross-sectional study
Source: Front Med (Lausanne). 2026 Oct 20;13:1973268. doi: 10.3389/fmed.2026.1973268 (PMC13727733; doi:10.3389/fmed.2026.1973268)
Supplement: Supplementary file 1 [file Table1.docx]

**Supplementary Table 1**. Baseline characteristics of the entire eligible cohort and the included study sample

| Variables | **Included in the study**  **(N=35,456)** | **Excluded from the study**  **(N = 165,205)** | Standardized mean difference |
| --- | --- | --- | --- |
| **Sociodemographic factors** |  |  |  |
| Age (year) † | 49.47±11.86 | 48.69±13.14 | 0.06 |
| Sex |  |  |  |
| Male | 13961 (39.38) | 59537 (36.04) | 0.07 |
| Female | 21495 (60.62) | 105667 (63.96) | -0.07 |
| Education level |  |  |  |
| ≤6 years | 914 (2.58) | 7885 (4.77) | -0.11 |
| 7-12 years | 10834 (30.56) | 55225 (33.44) | -0.06 |
| >12 years | 23708 (66.87) | 102052 (61.79) | 0.11 |
| Marriage |  |  |  |
| Unmarried | 6120 (17.26) | 35380 (21.43) | -0.10 |
| Married | 25338 (71.46) | 108081 (65.45) | 0.13 |
| Separated | 2715 (7.66) | 13861 (8.39) | -0.03 |
| Widowed | 1283 (3.62) | 7801 (4.72) | -0.05 |
| **Lifestyle behaviors** |  |  |  |
| Smoking |  |  |  |
| Never | 29256 (82.51) | 132499 (80.23) | 0.06 |
| Former | 3562 (10.05) | 16165 (9.79) | 0.01 |
| Current | 2638 (7.44) | 16479 (9.98) | -0.09 |
| Drinking |  |  |  |
| Never | 32104 (90.55) | 149136 (90.33) | 0.01 |
| Former | 961 (2.71) | 4786 (2.90) | -0.01 |
| Current | 2391 (6.74) | 11185 (6.77) | 0.00 |
| Physical activity |  |  |  |
| Inactive | 21286 (60.03) | 103176 (62.48) | -0.05 |
| Active | 14170 (39.97) | 61967 (37.52) | 0.05 |
| **Disease history** |  |  |  |
| Hypertension | 4186 (11.81) | 20579 (12.56) | -0.02 |
| Diabetes | 1855 (5.23) | 9501 (5.78)) | -0.02 |
| Hyperlipidemia | 2959 (8.35) | 13368 (8.16) | 0.01 |
| Stroke | 238 (0.67) | 1230 (0.75) | -0.01 |
| Valvular heart disease | 1577 (4.45) | 7003 (4.27) | 0.01 |
| Coronary artery disease | 490 (1.38) | 2424 (1.48) | -0.01 |
| Arrhythmia | 1638 (4.62) | 7448 (4.55) | 0.00 |
| Osteoporosis | 1482 (4.18) | 5875 (3.58) | 0.03 |
| Arthritis | 1789 (5.05) | 8353 (5.09) | 0.00 |
| Gout | 1428 (4.03) | 5950 (3.63) | -0.02 |
| Asthma | 1397 (3.94) | 7068 (4.30) | -0.02 |
| GERD | 5379 (15.17) | 24913 (15.16) | 0.00 |
| IBS | 1115 (3.14) | 4198 (2.55) | 0.04 |
| **Biomarker†** |  |  |  |
| BMI (kg/m2) | 24.24±3.84 | 24.32±4.02 | -0.02 |
| Waist (cm) | 83.46±10.42 | 83.33±10.92 | 0.01 |
| Hip (cm) | 96.35±7.24 | 96.22±7.48 | 0.02 |
| WHR | 0.86±0.07 | 0.86±0.07 | 0.00 |
| SBP (mmHg) | 118.71±17.92 | 120.31±18.07 | -0.09 |
| DBP (mmHg) | 73.83±11.27 | 73.91±10.92 | -0.01 |
| FPG (mg/dL) | 95.99±20.07 | 96.00±20.51 | 0.00 |
| HbA1c (%) | 5.76±0.78 | 5.76±0.79 | 0.00 |
| TG (mg/dL) | 114.88±92.79 | 114.97±97.45 | 0.00 |
| TC (mg/dL) | 196.34±36.30 | 195.84±37.58 | 0.01 |
| HDL-C (mg/dL) | 55.61±13.99 | 55.76±13.97 | -0.01 |
| LDL-C (mg/dL) | 120.24±32.04 | 119.54±32.36 | 0.02 |
| AST (U/L) | 22.86±11.89 | 23.00±13.52 | -0.01 |
| ALT(U/L) | 24.60±22.01 | 24.13±21.52 | 0.02 |
| SCr (mg/dL) | 0.74±0.67 | 0.72±0.32 | 0.05 |
| eGFR (ml/min/1.73m2) | 101.56±15.26 | 103.34±16.20 | -0.11 |

†: mean±SD; GERD: gastroesophageal reflux disease; IBS: irritable bowel syndrome; BMI: body mass index; WHR: waist-to-hip ratio; SBP: systolic blood pressure; DBP: diastolic blood pressure; FPG: fasting plasma glucose; HbA1c: hemoglobin A1c; TG: triglyceride; TC: total cholesterol; HDL-C: high-density lipoprotein-cholesterol; LDL: low-density lipoprotein; AST: aspartate aminotransferase; ALT: alanine aminotransferase; SCr: serum creatinine; eGFR: estimated glomerular filtration rate.

**Supplementary Table 2**. Associations of sex and body constitutions with lifestyle behavior as mediators after multiple imputation (n=200,661)

|  |  | OR (95% CI) |  | Proportion mediated (%)§ |
| --- | --- | --- | --- | --- |
| ***Outcome*** | Total effect† | Direct effect‡ | Indirect effect |  |
| **Yang deficiency** |  |  |  |  |
| Mediator: smoking | 1.34 (1.31, 1.37)*** | 1.37 (1.33, 1.40)*** | 0.98 (0.97, 0.99)*** | -8.00 (-11.63, -4.37)*** |
| Mediator: drinking | 1.36 (1.33, 1.39)*** | 1.37 (1.33, 1.40)*** | 1.00 (0.99, 1.00)*** | -1.45 (-2.31, -0.58)** |
| Mediator: physical inactivity | 1.38 (1.35, 1.41)*** | 1.37 (1.33, 1.40)*** | 1.01 (1.01, 1.01)*** | 4.06 (3.30, 4.81)*** |
| **Yin deficiency** |  |  |  |  |
| Mediator: smoking | 1.29 (1.26, 1.32)*** | 1.32 (1.29, 1.36)*** | 0.97 (0.96, 0.98)*** | -12.35 (-16.61, -8.10)*** |
| Mediator: drinking | 1.32 (1.29, 1.35)*** | 1.32 (1.29, 1.36)*** | 1.00 (1.00, 1.00) | -0.63 (-1.53, 0.28) |
| Mediator: physical inactivity | 1.34 (1.30, 1.37)*** | 1.32 (1.29, 1.36)*** | 1.01 (1.01, 1.01)*** | 3.65 (2.85, 4.46)*** |
| **Phlegm-stasis** |  |  |  |  |
| Mediator: smoking | 1.37 (1.34, 1.40)*** | 1.41 (1.38, 1.45)*** | 0.97 (0.96, 0.98)*** | -11.20 (-14.77, -7.64)*** |
| Mediator: drinking | 1.41 (1.37, 1.44)*** | 1.41 (1.38, 1.45)*** | 1.00 (1.00, 1.00)* | -0.80 (-1.58, -0.02)* |
| Mediator: physical inactivity | 1.43 (1.39, 1.47)*** | 1.41 (1.38, 1.45)*** | 1.01 (1.01, 1.01)*** | 4.10 (3.40, 4.80)*** |

*Percentile-based bootstrap CIs are reported.

**:p<0.01; ***:p<0.001.

†Adjusted for age, education, marriage, hypertension, diabetes, hyperlipidemia, stroke, valvular heart disease, coronary artery disease, osteoporosis, arthritis, gout, IBS, BMI, WHR, SBP, DBP, FPG, HbA1c, TC, TG, ALT, eGFR and sex.

‡Adjusted for age, education, marriage, hypertension, diabetes, hyperlipidemia, stroke, valvular heart disease, coronary artery disease, osteoporosis, arthritis, gout, IBS, BMI, WHR, SBP, DBP, FPG, HbA1c, TC, TG, ALT, eGFR sex and the mediators of interest (smoking, alcohol consumption or physical activity).

§The proportion mediated was calculated using the formula: (Natural indirect effect/Total effect)×100

**Supplementary Table 3**. Sensitivity analysis of the associations of sex and lifestyle behaviors with body constitutions using modified Poisson

|  | PR (95% CI) | | | |
| --- | --- | --- | --- | --- |
| Variables | Female  (male as reference) | Mediator: smoking  (never as reference) | Mediator: drinking  (never as reference) | Mediator: physical activity  (active as reference) |
| **Yang deficiency** |  |  |  |  |
| age-adjusted | 2.00 (1.91, 2.10)*** | 1.16 (1.09, 1.23)*** | 1.11 (1.04, 1.20)** | 1.34 (1.29, 1.40)*** |
| Multivariate-adjusted^1^ | 2.03 (1.93, 2.14)*** | 1.17 (1.10, 1.24)*** | 1.11 (1.04, 1.20)** | 1.35 (1.29, 1.41)*** |
| Multivariate-adjusted^2^ | 1.99 (1.88, 2.10)*** | 1.16 (1.09, 1.23)*** | 1.11 (1.03, 1.19)** | 1.34 (1.28, 1.40)*** |
| **Yin deficiency** |  |  |  |  |
| sex, age-adjusted | 1.64 (1.57, 1.72)*** | 1.22 (1.15, 1.29)*** | 1.07 (1.00, 1.15) | 1.32 (1.27, 1.38)*** |
| Multivariate-adjusted^1^ | 1.68 (1.60, 1.76)*** | 1.22 (1.16, 1.30)*** | 1.07 (1.00, 1.14) | 1.33 (1.27, 1.38)*** |
| Multivariate-adjusted^2^ | 1.72 (1.64, 1.82)*** | 1.21 (1.14, 1.28)*** | 1.06 (0.99, 1.14) | 1.31 (1.26, 1.37)*** |
| **Phlegm-stasis** |  |  |  |  |
| sex, age-adjusted | 2.16 (2.04, 2.29)*** | 1.29 (1.21, 1.39)*** | 1.14 (1.04, 1.24)** | 1.49 (1.42, 1.57)*** |
| Multivariate-adjusted^1^ | 2.23 (2.10, 2.37)*** | 1.31 (1.22, 1.40)*** | 1.13 (1.04, 1.23)** | 1.50 (1.42, 1.58)*** |
| Multivariate-adjusted^2^ | 2.39 (2.23, 2.55)*** | 1.28 (1.19, 1.37)*** | 1.12 (1.03, 1.22)** | 1.45 (1.38, 1.53)*** |

**:p<0.01; ***:p<0.001

Data are presented as prevalence ratios (95% Confidence Interval).

Multivariate-adjustment^1^ for age, education, marriage, smoking, alcohol consumption, leisure-time physical activity, hypertension, diabetes, hyperlipidemia, stroke, valvular heart disease, coronary artery disease, osteoporosis, arthritis, gout, and IBS.

Multivariate-adjusted^2^ for age, education, marriage, smoking, alcohol consumption, leisure-time physical activity, hypertension, diabetes, hyperlipidemia, stroke, valvular heart disease, coronary artery disease, osteoporosis, arthritis, gout, IBS, BMI, WHR, SBP, DBP, FPG, HbA1c, TC, TG, ALT, and eGFR.

b

a

c’

c

Sex

**Lifestyle behaviors**

**TCM body constitution**

*Indirect association: c-c’=ab*

*Direct association: c’=c-ab*

**Supplementary Figure 1.** A model of the mediating effect of lifestyle behaviors on the relationship between sex and TCM body constitution
